# Efficacy of LED Photobiomodulation for Functional and Axonal Regeneration After Facial Nerve Section-Suture

**DOI:** 10.3389/fneur.2022.827218

**Published:** 2022-02-23

**Authors:** Hafsa Er-Rouassi, Luc Benichou, Badiaa Lyoussi, Catherine Vidal

**Affiliations:** ^1^Centre Borelli, CNRS UMR-9010, Université de Paris, Paris, France; ^2^Laboratory of Natural Substances, Pharmacology, Environment, Modeling, Health, and Quality of Life (SNAMOPEQ), Department of Biology, Faculty of Sciences Dhar Mehraz, Sidi Mohamed Ben Abdellah University, Fez, Morocco; ^3^Paris-Est Créteil Université (UPEC) Faculté de Médecine, Creteil, France

**Keywords:** buccal and marginal mandibular branches of facial nerve, distal pes, infrared therapy, photobiomodulation, nerve regeneration, fluorogold retrograde labeling, nasal deviation, eyelid closure

## Abstract

Facial nerve damage can lead to partial or total facial nerve palsy. Photobiomodulation has been reported to improve and accelerate functional recovery following peripheral nerve lesion, depending on the type of lesion and the light exposure parameters used. The aim of this study was to investigate the effects of infrared exposure on functional and axonal regeneration after section-suture of the distal branches of the facial nerve: the buccal and marginal mandibular branches and the distal pes. The animals underwent surgery and were irradiated with infrared light at 850 nm twice daily from day 1 to day 16. The recovery of facial function was then studied at both the behavioral and morphological levels. Behavioral analyses were performed by videoscoring with a high-speed camera and using various devices to assess the recovery of whisker movement on the lesioned side from day 1 to day 30. We also assessed nasal deviation toward the intact side and the ability to close the ipsilateral eyelid completely from day 1 to day 38 and from day 1 to day 50, respectively. For morphological analyses, we assessed the re-establishment of facial motoneuron labeling with Fluorogold®, an immunofluorescent retrograde marker of axonal transport injected into the vibrissae, on D10, D14 and D30. We found that whisker movements recovery was significantly faster in treated than in control mice. A complete disappearance of nasal deviation was observed at 2 weeks in infrared-treated lesioned mice and at 5 weeks in controls. Complete eyelid closure was observed 3 weeks after surgery in treated animals and 6 weeks after surgery in controls. Finally, normal fluorogold labeling of the facial nuclei complex was restored 30 days after surgery in the treated animals, but no such restoration was ever observed in control animals. In conclusion, our data show that IR treatment at a distal site has a significant positive effect on facial nerve recovery. These findings pave the way for the clinical use of infrared photobiomodulation in patients with nerve lesions.

## Introduction

Peripheral nerve lesions cause major motor and/or sensory disorders that can significantly impair quality of life. Facial palsy, for example, is a severe handicap with severe consequences for the patient's professional, social and family life.

Damaged peripheral nerves can regenerate, but they often do so imperfectly, resulting in very difficult living conditions for the injured patients. The annual incidence of facial palsy is about 15 to 30 cases per 100,000 people. Bell's palsy accounts for 80% of these cases. It occurs suddenly, generally improving after a few weeks, but sometimes with sensory or motor sequelae. In 20% of cases, the origin is traumatic, vascular, post-surgical, or even cerebellopontine angle tumors, such as vestibular schwannoma. Numerous studies have been performed with the goal of enhancing and accelerating the functional recovery of such peripheral nerve lesions (1–4).

The facial nerve is an excellent model (5) for studies of post-lesion peripheral neuronal plasticity for three reasons: 1) its anatomy has been described in detail; 2) motor outputs (movements of the vibrissae and blink reflexes) are easy to quantify, and 3) the facial nuclei are well described and easily accessible in rodents *in vivo* and on brain sections *in vitro* for electrophysiological and morphological studies.

In cases of neurological injuries or neurotmesis lesions according to the Sunderland classification (6), in which the nerve has undergone a complete sectioning of the axon and its surroundings, surgical repair may be beneficial. End-to-end epineural neurorrhaphy is a “standard technique” for bringing the stumps of the nerve together after a complete section. The stumps can be brought together without excessive tension and then sutured, increasing the chances of recovery (7).

Photobiomodulation (PBM) is a type of treatment with light of visible and infrared (IR) wavelengths. These waves provide the cells with metabolic energy and adjust their functioning in terms of cell and molecular biology. Within the cells, this energy activates cytochrome *c* in the mitochondria, leading to the production of adenosine triphosphate (ATP), which activates the microcirculation and triggers the production of nitric oxide (NO) and cell growth factors (8). As a result, the expression of various genes relating to many cell functions is modulated, leading to cell proliferation, cell migration, lower cell death rates, and enhanced neurological function (9).

Many recent studies investigating the effects of PBM therapy in peripheral nerve injury models have shown an immediate protective effect, boosting electrical activity in the injured peripheral nerve (10, 11). PBM therapy also modulates inflammatory processes, by reducing edema formation and inflammatory cell migration (12). Finally, IR irradiation has been shown to increase the rate of axonal growth and the thickness of myelin sheaths (13).

The objective of this study was to evaluate the effect of PBM on the functional recovery and axonal regeneration of the facial nerve after distal or proximal unilateral section-suture in SN-SWISS-M mice. We had three major aims:

- To develop a reliable model of facial nerve lesions at the level of the buccal and mandibular branches or the pes distal branch in Swiss mice.
- To analyze the behavioral effect of IR phototherapy on whisker movement, nasal deviation and eye closure.
- To analyze the recovery of facial motoneuron retrograde fluorogold (FG) labeling in treated and control mice after distal section-suture of the facial nerve.

## Methods

### Animals and Experimental Design

The experimental procedures performed were approved by the Ethics Committee for Animal Experimentation of the University of Paris in accordance with the requirements of the European Communities Council Directive of September 22, 2010 (2010/63/UE). We used 184 two-month-old male Swiss mice, each weighing 30–35 g, in this study. All animals were kept in appropriate boxes, at 22°C and were supplied with water and food *ad libitum*.

We used three different models in this study:

1) Distal BM section-suture (SS): the buccal and marginal mandibular (BM) branches of the left facial nerve were sectioned approximately 20 mm distal to the main trunk at the exit from the stylomastoid foramen. End-to-end epineural suture was then performed.
2) Distal pes SS: as for model one but with section-suture performed at the level of the pes (a convergence of the buccal and marginal mandibular branches just proximal to the whisker pad) in order to obtain a faster recovery than the first model in controls (Distal BM SS).
3) Proximal B SS: a proximal section-suture of the buccal branch (B) was performed 10 mm distal to the main trunk of the facial nerve in order to evaluate the effect of IR treatment on the eyelid closure.

The animals were split into the groups illustrated in Table 1. In the control group, the facial nerve was injured but no treatment was administered. In the IR-treated group, the injured facial nerve was treated by IR light exposure. In the red-treated group, the injured facial nerve was treated by exposure to red light, and in the sham group, the facial nerve was injured, and the mice were exposed daily (for 16 days) to isoflurane anesthesia without treatment.

**Table 1:** Experimental design.

**Surgical models**			**Behavioral study**	**Retrograde fluorogold labeling**		
			**D1 to D30**	**D10**	**D14**	**D30**
**Distal BM SS**	Control group		*n =* 12	*n =* 12	*n =* 12	*n =* 12
	IR-treated group	At the facial nerve lesion	*n =* 12	*n =* 12	*n =* 12	*n =* 12
		At the facial nerve lesion and the ipsilateral whiskerpad	*n =* 6	-	-	-
		At the ipsilateral whisker pad only	*n =* 6	-	-	-
	Red light-treated group	At the facial nerve lesion	*n =* 16	-	-	-
	Sham group		*n =* 8	-	-	-
**Distal pes SS**	Control group		*n =* 12	-	-	*n =* 6
	IR-treated group	At the facial nerve lesion	*n =* 12	-	-	*n =* 6
**Proximal B SS**	Control group		*n =* 8	-	-	-
	IR-treated group	At the facial nerve lesion	*n =* 8	-	-	-

*Distal BM section-suture was performed on a control group, an IR-treated group, a red light-treated group and a sham group. Distal pes section-suture was performed on control and IR-treated groups. Similarly, proximal section-suture of the buccal branch was performed on control and IR-treated groups. For the behavioral study, the number of mice used is indicated for each group. Similarly, in the morphological study the number of mice is indicated for D10, D14 and D30*.

### Facial Nerve Surgery

All animals were weighted and placed under general anesthesia for surgery. Animals were anesthetized with intraperitoneally administered ketamine 1000 (Vibrac France, 100 mg/ml) and xylazine (Rompun 2%). The head of the animal was shaved, and an incision was made in the skin of the face with a scalpel blade. This incision extended approximately from the tragus of the ear to the start of the whisker pad on the left side.

#### Distal SS of the Buccal and Marginal Mandibular Branches Model (Distal BM SS)

The buccal and marginal mandibular branches of the facial nerve were exposed, and the parotid gland (the superficial part) was removed. The two branches (buccal and marginal mandibular) were sectioned with a fine straight-tipped surgical scissor (Moria SAV, France) under a light microscope. End-to-end epineural suture was then performed with 10/0 monofilament nylon sutures (Ethicon; Johnson & Johnson, France) to connect the distal and proximal stumps of each branch (Ethicon; Johnson & Johnson, France). The surgical procedure was completed by suturing the skin with 5-0 silk thread (Ethicon; Johnson & Johnson, France). Note that the sectioning of a single branch (buccal or marginal mandibular), does not cause complete whisker paralysis in Swiss mice.

#### SS of the Distal Pes (Distal SS Pes)

A smaller incision was made immediately proximal to the whisker pad, and the same surgical procedure as for the first model was performed on the distal pes (which is the convergence of the buccal and marginal mandibular branches just proximal to whisker pad) (14).

#### Proximal SS of the Buccal Branch (Proximal SS B)

The buccal ramus was sectioned about 10 mm distal to the main trunk of the facial nerve at its exit from stylomastoid foramen and sutured under the same conditions as for the first model, to induce isolated ipsilateral eyelid palsy.

### Photobiomodulation Therapy

The animals in the treated groups were subjected to PBM therapy twice daily (5 days in every 7) for 16 days. Sham and treated mice were anesthetized slightly with volatile isoflurane anesthesia (initiation at 2–3%, oxygen flow rate 0.8–1.5 l/min and maintenance at 1.5% oxygen flow 500ml/min) before each session (anesthesia duration 3 min). In the treated group, a LED prototype (Qlarité HK) was placed in direct contact with the skin surface, perpendicular to the facial nerve lesion, and light pulses were applied with the following parameters: wavelength of 850 ± 10 nm, optical power output potency of 75 mW, 50 Hz pulse mode, power density of 570 mW/cm^2^, beam area of 0.13 cm^2^, exposure time of 240 s per day and a total energy per session of 9 Joules (see Table 2). The interval between each session a day was 6 h, i.e., 10 am 16 pm. Finally, light treatment began one day after surgery (D1).

**Table 2:** IR light parameters.

Power	75 mW
Power density	577 mW/cm^2^
Energy density	69.23 J/cm^2^
Wavelength	850 nm ± 10 nm
Application time	240 s per day
Wave type	Pulsed wave (PW)
Frequency	50 Hz
Duty cycle	50%
Beam direction	Perpendicular to the suture
Energy	9 J
Spot area	0.13 cm^2^
Number of days	16

The 9 J pulsed mode was chosen according to the results obtained from our dose-response curve.

We used three different protocols of irradiation in the distal BM SS:

- One with irradiation at the level of the skin above the SS.
- Another with irradiation at the level of the skin above the SS plus irradiation of the ipsilateral whiskerpad.
- Irradiation of the whiskerpad only.

In the pes model, these protocols for irradiation of the muscles in not necessary since the placement of the LED prototype delivering IR had the advantage to irradiate both whiskerpad and the SS facial nerve.

The effectiveness of double PBM treatment, applied at two areas: injured area of the facial nerve, and corresponding denervated muscle (ipsilateral whiskerpads), was evaluated with the goal for restoring denervated muscle atrophy and achieving improved vibrissae function. The mice in the sham group were anesthetized with isoflurane every day for 16 days, without treatment.

### Measurement of Facial Nerve Function

Facial nerve function was evaluated by monitoring the movements of the whiskers, nasal deviation and eyelid closure. All measurements were performed by two independent observers blind to treatment.

#### Whisker Movements

The mice were subjected to a training session 1 week before surgery. The movements of their whiskers were filmed with three different devices and a high-speed video camera (Sony), operating at a rate of 100 frames per second. Each session lasted 2–5 min. The animals were placed on different devices:

1) Rotarod: we assessed the ability of the mice to balance on a bar turning at constant speed (four turns per minute). Whisker movements were recorded on a film over a period of 2–5 min. The ears of the mice were in the forward position throughout, indicating that the animals were not stressed.
2) Perspex bar: in this test, the animals had to pass from point A to point B (covering 39.5 cm) for the measurement of whisker movements during active locomotion. The mice frequently stopped in the middle and looked down, moving their left and right whiskers together. This made it possible for us to film the heads of the mice from above (15) and to analyze the amplitude of protraction-retraction for the left and right whiskers together.
3) Dark tunnel: The mice were placed in a small dark tunnel (6 cm long). They remained calm within the tunnel, performing very few head movements, facilitating the recording of very clear whisker movements.

Two experimenters blind to group performed computer analyses of the films on D1, D3, D7, D10, D14, D16, D25, D30, and D60 after surgery. Scores were attributed as previously described (16), but with a slight adjustment of the classical scores according to whisker movements in the different devices, based on intermediate scores (Table 3). A precise evaluation of protraction movements was performed by video capture system (NCH Videopad software), making it possible to determine the complete amplitude recovery of the protraction. In rat, the amplitude of the movement from maximum protraction (62.0° ± 13.2) to maximum retraction is about (57° ± 13) (2).

**Table 3:** Videoscoring of whisker movement recovery—this scoring system differs from published scores because we took into account the separate scores obtained for each device to obtain a final score.

**Rota-rod score**	**Bar score**	**Head-down score**	**Tunnel score**	**Final score**
0	0	0	0	0: No movement of the whiskers on the lesioned side
1	1	1	1	1: Slight fibrillations on the injured side relative to the intact side
2	2	1	2	1.5: Slight fibrillations on the injured side relative to the intact side for some but not all devices
2	2	2	2	2: Significant (but asymmetric) voluntary movement of the lesioned side relative to the intact side (amplitude)
3	3	2	3	2.5: Significant (but asymmetric) voluntary movement of the lesioned side relative to the intact side for some, but not all devices
3	3	3	3	3: Symmetric amplitude and frequency of voluntary whisker movement on both sides

*The amplitude of whisker protraction was studied in detail, particularly in the third row (C3) “head-down” HD*.

#### Measurement of Nasal Deviation

A frontal photograph of each animal was taken at baseline, before surgery. Two experimenters then took frontal photographs on the surgery table or in the tunnel, for the animals of each group, on D1, D3, D7, D10, D14, D16, D25, D30, D35, and D38 after surgery.

**Figure 1:**
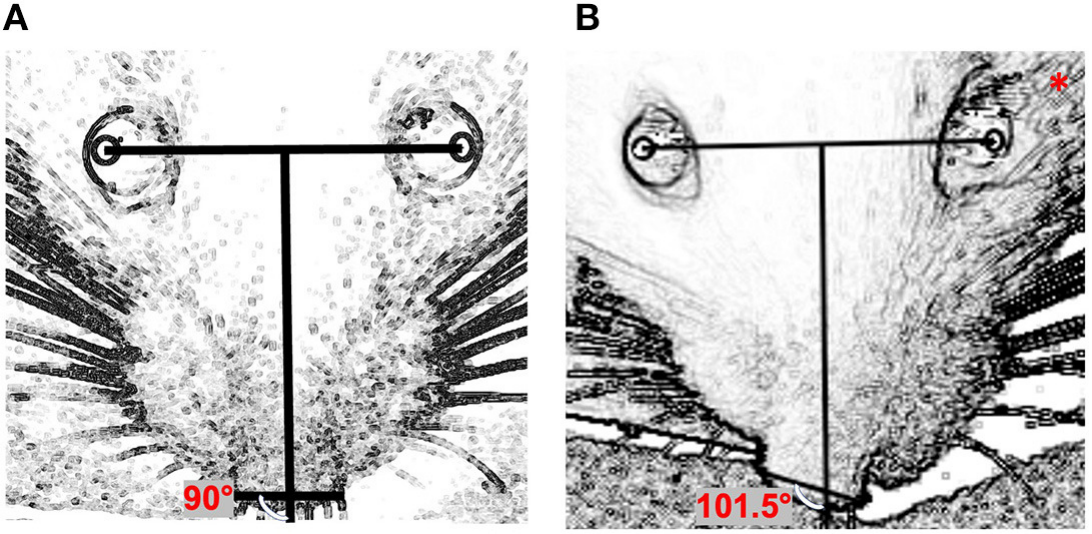
Quantification of nasal deviation: **(A)** preoperative day (normal nasal position); **(B)** one day after surgery (left side); *side on which surgery was performed. The angle between the perpendicular line with the interpupillary horizontal line and the line connecting the two nostrils was used for quantification.

Image J software was used to determine the changes in nasal position as follows (Figure 1): a line (the “inter-iris line”) was drawn linking the center of the pupil of the right eye and the center of the pupil of the left eye. A second line, perpendicular to the first, was then drawn midway between the irises. A third line was added connecting the outer edges of the two nostrils. The angles between the line perpendicular to the inter-iris line and the line connecting the nostrils were measured.

#### Eyelid Closure

Eyelid closure is controlled by the orbicularis oculi muscle (OOM), which is innervated by the zygomatic branch (buccal ramus) of the facial nerve. We stimulated the cornea on the injured side with a wet cottonwool ball at the extremity of a thin metallic rod. This induces an eyelid closure reflex in normal mice, but not in mice that have undergone proximal buccal SS. Eyelid closure was scored as follows: 0: no movement; 0.5: incomplete eyelid closure; 1: complete eyelid closure.

### Re-innervation Assessment

Retrograde tracing techniques were used to assess in detail the connections between a target population of neurons (facial motoneuron: FMNs) and the innervating muscles (whiskerpads). We injected 20 μL Fluorogold© (1% in saline; Vector Laboratories, France), a retrograde fluorescent marker, subcutaneously into the contralateral and ipsilateral whisker pads (C3 vibrissae, the third line of vibrissae in the third row) of mice under general anesthesia. Injections were performed on D8, D12, and D28 for the distal BM SS model and on D28 for the distal pes SS model. No injection was performed on D14 for the distal pes SS model, due to the diffusion of Fluorogold© and variability in the number of FG-labeled motoneurons.

**Figure 2:**
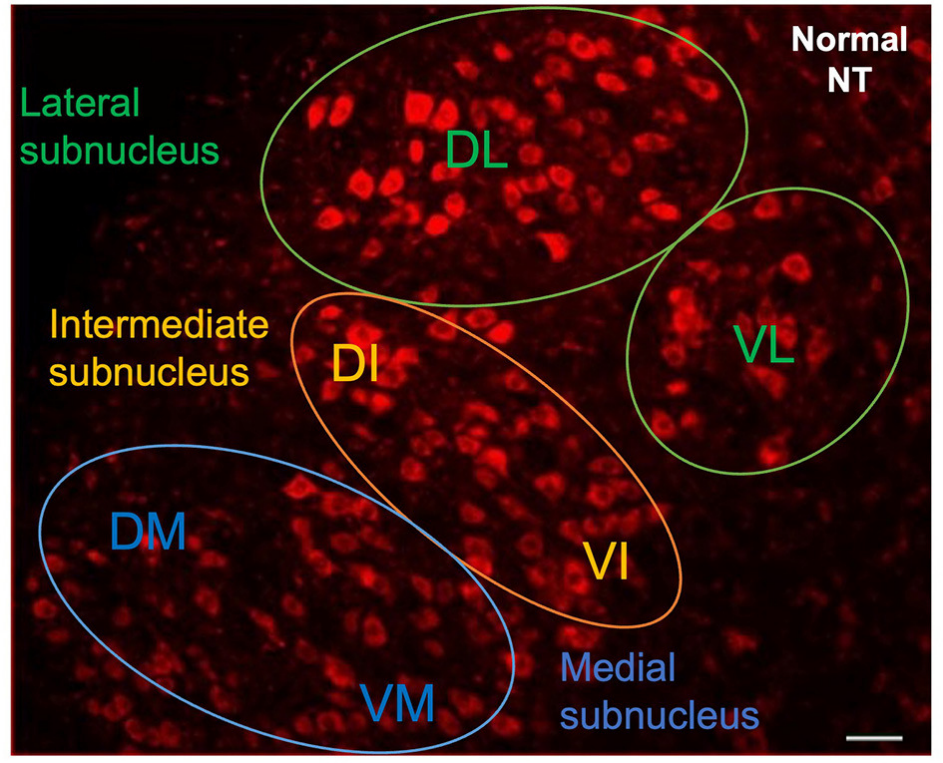
Neurotrace counterstaining of facial motoneurons in contralateral side following injection into whiskerpads. Letters indicate the names of subnuclei: VL, ventrolateral; DL, dorsolateral; DI, dorsal intermediate; VI, ventral intermediate; DM, dorsomedial; VM, ventromedial. Scale bars = 20 μm.

The animals were deeply anesthetized 48 h later, and trans-cardially perfused with 4% paraformaldehyde. The whole brain was removed and post-fixed by incubation overnight in the same solution 4% paraformaldehyde (PFA) at 4°C, 2 days before rehydration with phosphate buffered saline (PBS). Two blocks from the middle of the brain corresponding to the right and left facial complexes were sectioned in the sagittal plane. We cut serial 30 μm sections from the ventrolateral part of the brainstem with a vibratome (LEICA VT1000 S, France). The sections were counterstained with NeurotraceTM 530/615 Red Fluorescent Nissl Stain (Thermo Fisher Scientific, France). They were mounted in Vectashield (Vector Laboratories, France) antifade mounting medium and observed under a fluorescence microscope (NIKON E800). The images were then digitized with a computerized image analysis system (Metaview, Rupper Scientific, France). The number of motoneurons labeled with FG was quantified with Image J software (version 1.52). We counted the labeled motoneurons in the lateral and intermediate nuclei of each section according to a well-defined protocol: only cells displaying intermediate or intense FG labeling with a diameter of at least 20 μm were counted. Motoneurons were counted from all consecutive sections. Corrections were made for double counting according to the method of Abercrombie (17). The Neurotrace (NT) counterstaining was used to identify the location of the FG-labeled motoneurons in the various subnuclei of the facial complex (Figure 2).

### Statistical Analysis

The whisker movement and blink reflex data were analyzed by one-way analysis of variance (one-way ANOVA). Nasal deviation and the total number of regenerated motoneurons were analyzed with unpaired *t*-tests (*p* < 0.05 considered significant). GraphPad prism 8.0 / xlstat was used for statistical analysis.

## Results

We studied first the dose-response relationship between PBM and facial nerve functional recovery. We identified the best protocol for IR irradiation based on our results. We then studied the functional and neuronal recovery of the injured facial nerve over time.

### Dose-Response Relationship for PBM

We evaluated the effects of IR light at different energies on the functional recovery of the facial nerve after distal section-suture injury. We used an 850 nm LED with a power of 75 mW and variable energy for PBM. Seven experimental groups were studied (*n* = 6 in each group): a distal section-suture BM group without treatment, and six groups received PBM at various energies (doses): 2.25–4.5–9–18–45 and 90 J (corresponding to 1, 2, 4, 8, 10, and 20 min). From 2.25 to 18 J, irradiation was delivered as pulses, whereas, for the last two energies (45, 90 J), the irradiation was delivered in continuous mode.

Low-energy treatment, at 2.25 or 4.5 J, had no effect on functional recovery (no difference with respect to the control group). Faster whisker movement recovery on the lesioned side was observed after treatment at 9 J, with no difference detected between 9 and 18 J. Treatment at higher energies had neither a positive nor a negative effect relative to untreated animals. Pulsed-mode irradiation gave much better overall results than continuous irradiation. Finally, we found no negative effect at high intensities (90 J).

### Behavioral Results: Effects of IR on Functional Recovery on the Lesioned Side in the Three Surgical Models

#### Whisker Movements

A similar recovery of whisker movements was observed in both models (distal BM SS and distal pes SS). On D0, the whiskers on the injured side were immobile and caudally retracted, in all the mice that had undergone surgery. Whisker retraction and a total absence of whisker movement were essential to demonstrate the effective complete sectioning of both the buccal and marginal mandibular branches. Retraction persisted for about 3 days. All the animals of the distal SS BM group had a score of 1, corresponding to fibrillations, at D6. Similar results were obtained for the animals treated with IR.

**Figure 3:**
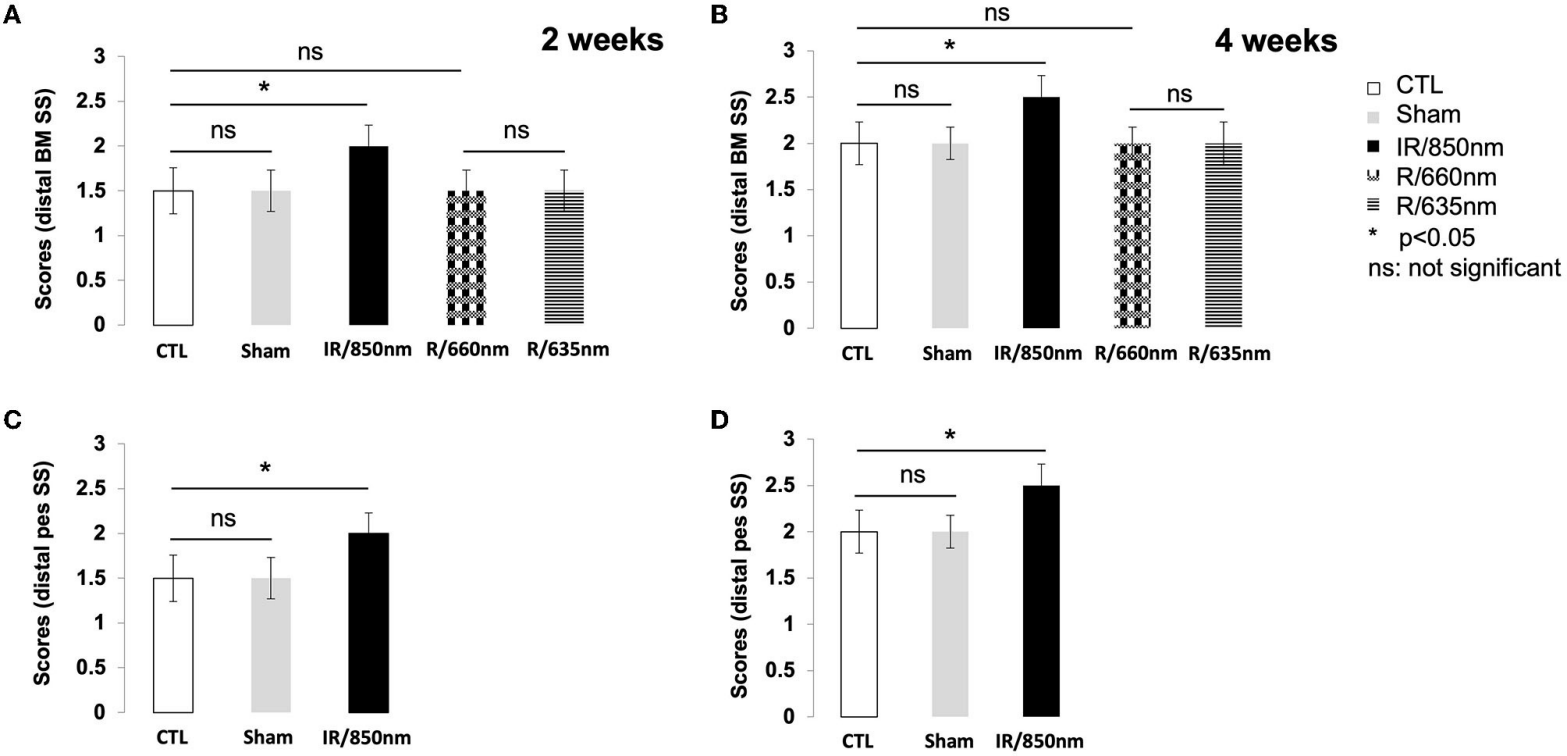
Graph of scores for whisker movements. Functional results 2 and 4 weeks after surgery. **(A)** Whisker movements after distal section-suture of the buccal and marginal mandibular branches in the different groups [control (*n* = 12): operated untreated group, sham (*n* = 8): operated untreated group exposed to isoflurane anesthesia (*n* = 12), IR treated group (*n* = 12) and red treatment group (*n* = 16), with treatments at two different wavelengths] two weeks after surgery. A score of 2 was recovered only in the IR group (*p* < 0.05). **(B)** Whisker movements after distal section-suture of the buccal and marginal mandibular branches in the different groups four weeks after surgery. A score of 2.5 was recovered at day 30 only in the group treated with IR (*p* < 0.05). **(C)** Whisker movements after section-suture of the distal pes in the control group (*n* = 12) and IR treated group (*n* = 12) two weeks after surgery. A score of 2 was recovered only for the treated mice (*p* < 0.05). **(D)** Whisker movements after section-suture of the distal pes in the control group and the IR treatment group two weeks after surgery and four weeks later. A score of 2.5 was recovered at day 30 only in the group treated with IR (*p* < 0.05). [*(*p* < 0.05); one-way ANOVA].

Two weeks after surgery, a score of 2 was recorded for all the treated mice but none of the animals in the control group. The untreated mice did not achieve a score of 2 until D21. There was no significant difference between the distal SS control and red light-treated groups (Figure 3A). Similar findings were obtained for the sham group, which did not differ significantly from the control group (*P* > 0.05).

Four weeks after surgery, active movements on the lesioned and intact sides were observed in all the mice treated with IR but none of the untreated animals. However, protraction movements were weaker on the ipsilateral side than on the contralateral side. The maximum score for whisker movements was therefore 2.5, rather than 3, because of the incomplete recovery of protraction movements. We investigated the possible disappearance of this deficit with time after injury, by allowing the animals to recover for an additional 8 weeks (day 60). No improvement in the amplitude of protraction was observed on the injured side (Figure 3B). Double irradiation of the ipsilateral vibrissae and skin surface over the SS of the nerve did not improve the score at D30. The Figures 3C,D illustrate the recovery of movements in distal pes SS model 2 and 4 weeks post-surgery.

#### Nasal Deviation

**Figure 4:**
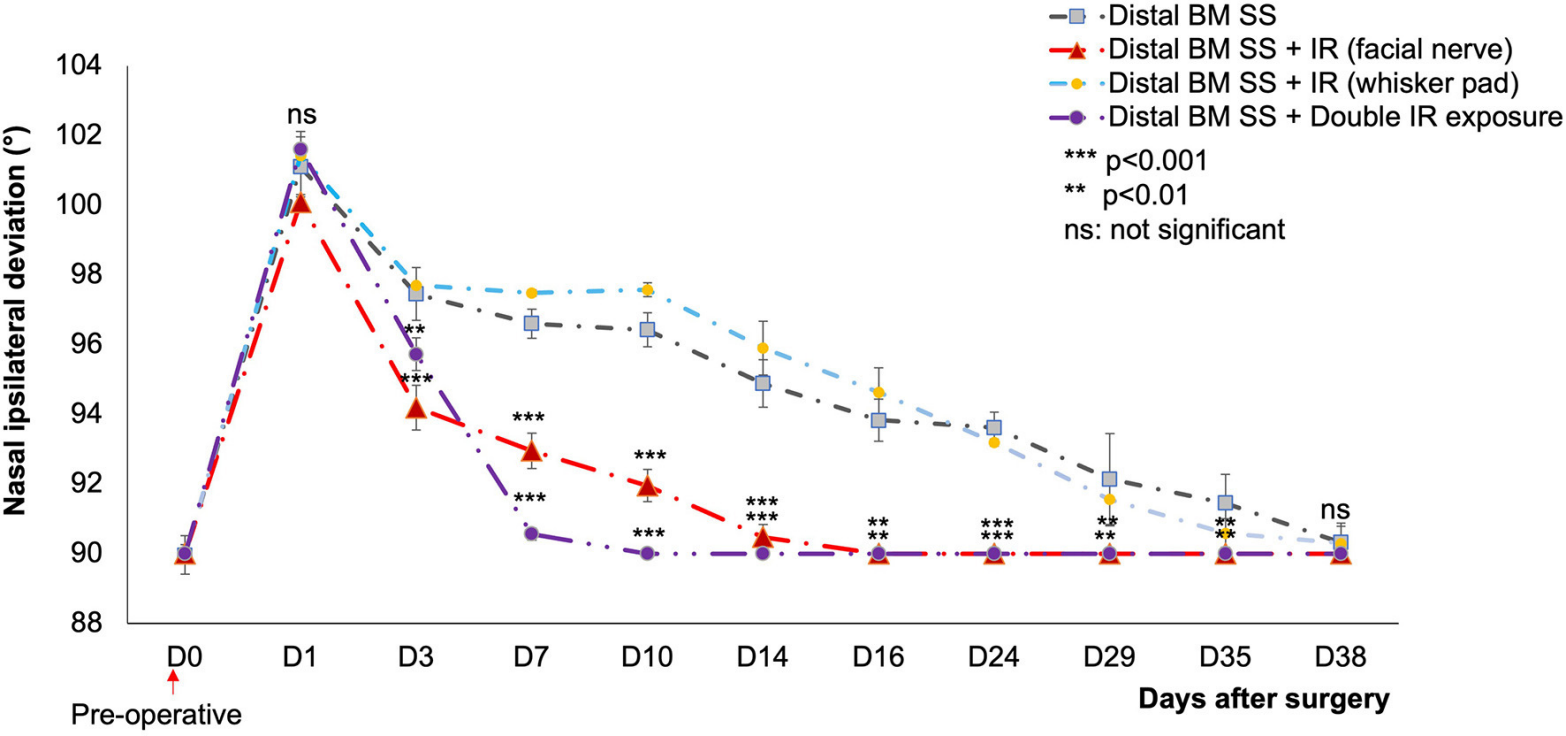
Mean nasal deviation (°) in the different groups, for D0 to D38. The initial nasal position was recovered on day 7 (double IR exposure) and D16 (single IR exposure) only for IR-treated mice (*p* < 0.001) and (*p* < 0.01) respectively compared to control. No significant difference was observed between the control and the IR treated group (at the ipsilateral whisker pad) (*p* > 0.05). 
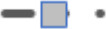
: distal SS of the buccal and marginal mandibular branches without treatment (*n* = 12). 
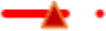
: distal SS of the buccal and marginal mandibular branches with IR treatment at one site (facial nerve suture) (*n* = 12). 
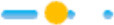
: injured nerve (distal SS of the buccal and marginal mandibular branches) with IR treatment at the ipsilateral whisker pad (*n* = 6). 
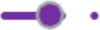
: injured nerve (distal SS of the buccal and marginal mandibular branches) with two irradiated areas: (1) injured area of the facial nerve, and (2) corresponding whisker pads on the ipsilateral side. Distal Pes SS: injured nerve without treatment (*n* = 6). [**(*p* < 0.01) ***(*p* < 0.001); *t* test].

A strong nasal deviation to the intact side was observed on D1 in both control and treated mice. This deviation persisted until D35 in the controls, but not in the treated groups. For animals subject to IR treatment as a single exposure of the skin above the lesioned nerve, a significant decrease in nasal deviation was observed at D16 (*p* < 0.01), with a return to the normal position (Figure 4).

Following double irradiation (irradiation of both the lesioned side and the denervated whisker pad), the mice recovered their initial nasal position on D7 (*p* < 0.001). No significant difference was observed between the two surgical techniques.

#### Eyelid Closure

**Figure 5:**
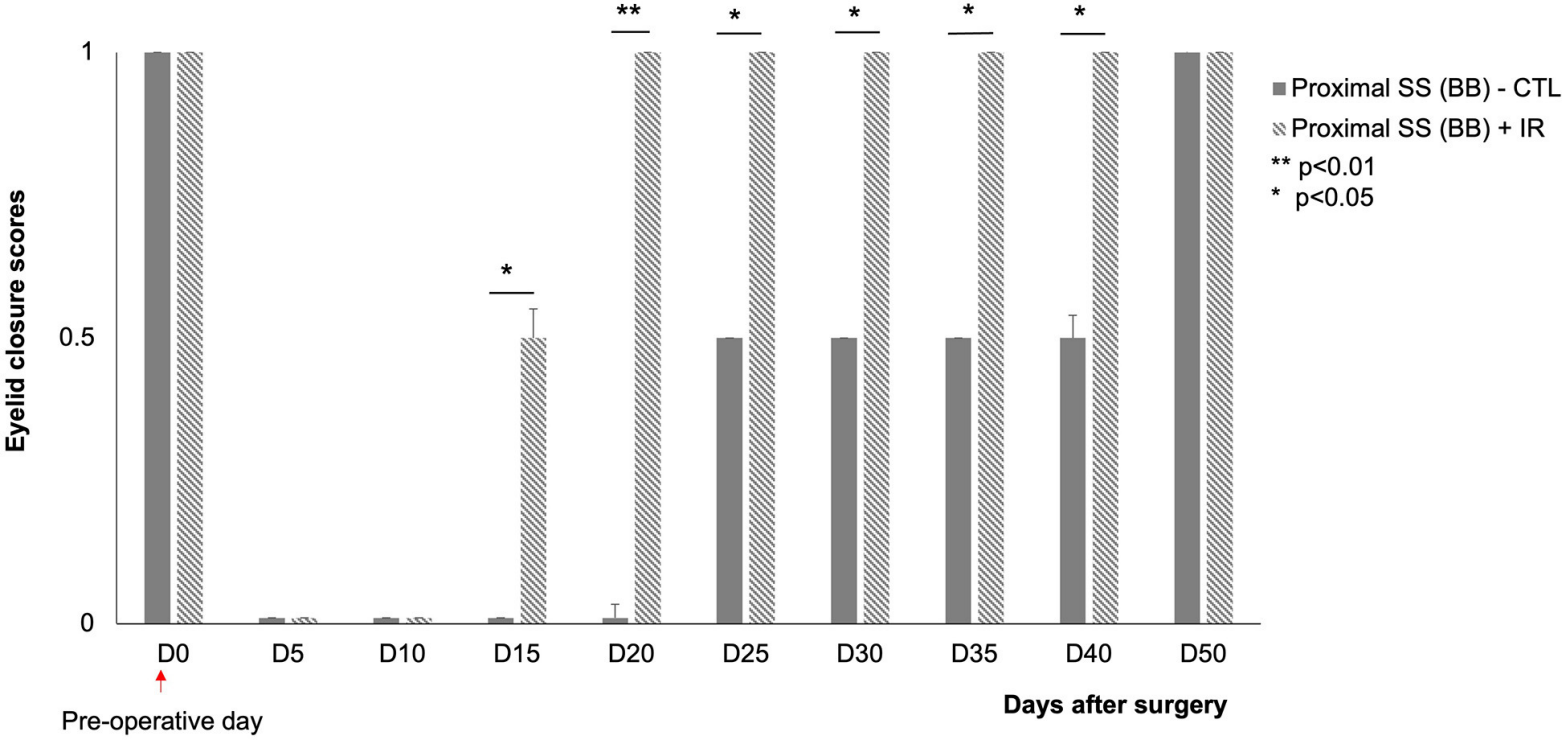
Scores for eyelid closure on the injured side (left) at various time points after surgery. 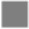: animals subjected to proximal section-suture of the buccal branch without treatment (*n* = 8), 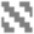: injured nerve (proximal section-suture of the buccal branch) with IR treatment (*n* = 8). Note the significant difference between treated and untreated mice (complete recovery of eyelid closure at D20 for treated mice and D50 for untreated mice). [*(*p* < 0.05) **(*p* < 0.01); one-way ANOVA].

A proximal section-suture of the buccal branch results in an initial loss of spontaneous eye closure suppress in response to corneal stimulation. The control animals were unable to close their eyelids until D50 after surgery. The mice exposed to IR treatment regained the ability to close their eyes almost completely within 20 days after surgery (Figure 5).

### Morphological Study: Retrograde FG Labeling of Neurons

The facial motoneurons with axons extending were evaluated for FG labeling. Following Fluorogold© injection into the whisker pads, most of the labeled motoneurons were found in lateral positions within the facial complex, particularly in the lateral subnuclei, although some labeled cells were observed in the dorsal portion of the intermediate subnuclei.

**Figure 6:**
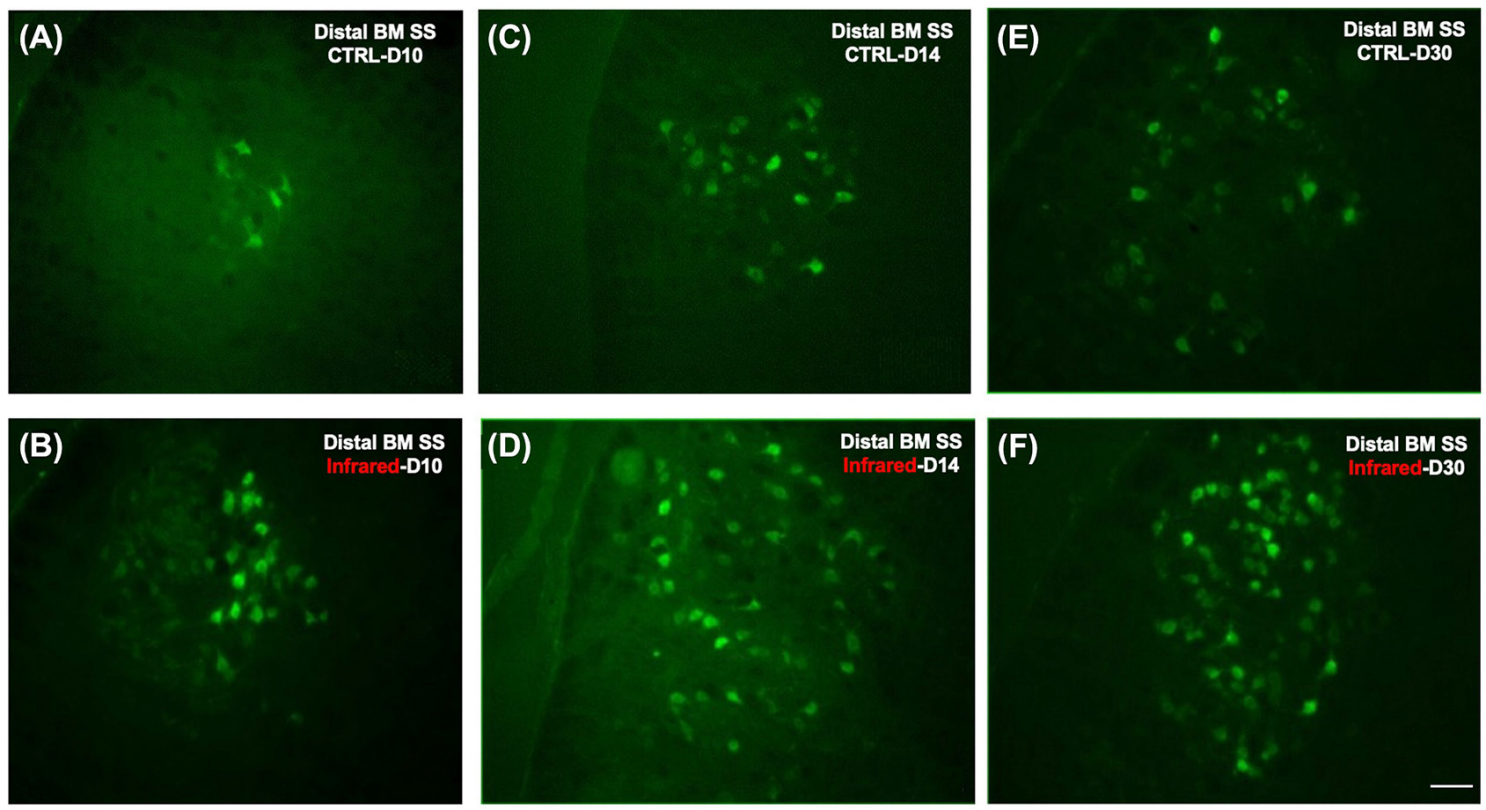
Fluorescence photomicrographs of motoneurons retrogradely labeled with Fluorogold on the ipsilateral side after section-suture of the buccal and marginal mandibular branches. **(A)**: Distal BM SS - control group at D10 (*n* = 12). Note that only a few cells are labeled. **(B)**: Distal BM SS - IR-treated group at D10 (*n* = 12). Note that a few cells are labeled, as in the control group. **(C)**: Distal BM SS - control group at D14 (*n* = 12). Note that the number of labeled cells is greater than at D10, although it remains low. **(D)**: Distal BM SS - IR-treated group at D14 (*n* = 12). Note that the number of labeled motoneurons is greater than that in the control group. **(E)**: Distal BM SS - control group at D30 (*n* = 12). Note that the number of labeled facial motoneurons has increased. **(F)**: Distal BM SS - IR-treated group at D30 (*n* = 12). Note the larger number of FG-labeled motoneurons on the lesioned side of treated mice than on that of untreated mice **(E,F)**. Scale bars = 20 μm.

Figure 6 shows the labeled facial motoneurons in the brainstem of control and lesioned mice at various times after surgery. Only a few facial motoneurons were labeled in the controls and in IR-treated mice (Figures 6A,B). At D14 and D30 after surgery, the number of labeled facial motoneurons was increased in the IR-treated mice group than in the control group (Figures 6C–F).

**Figure 7:**
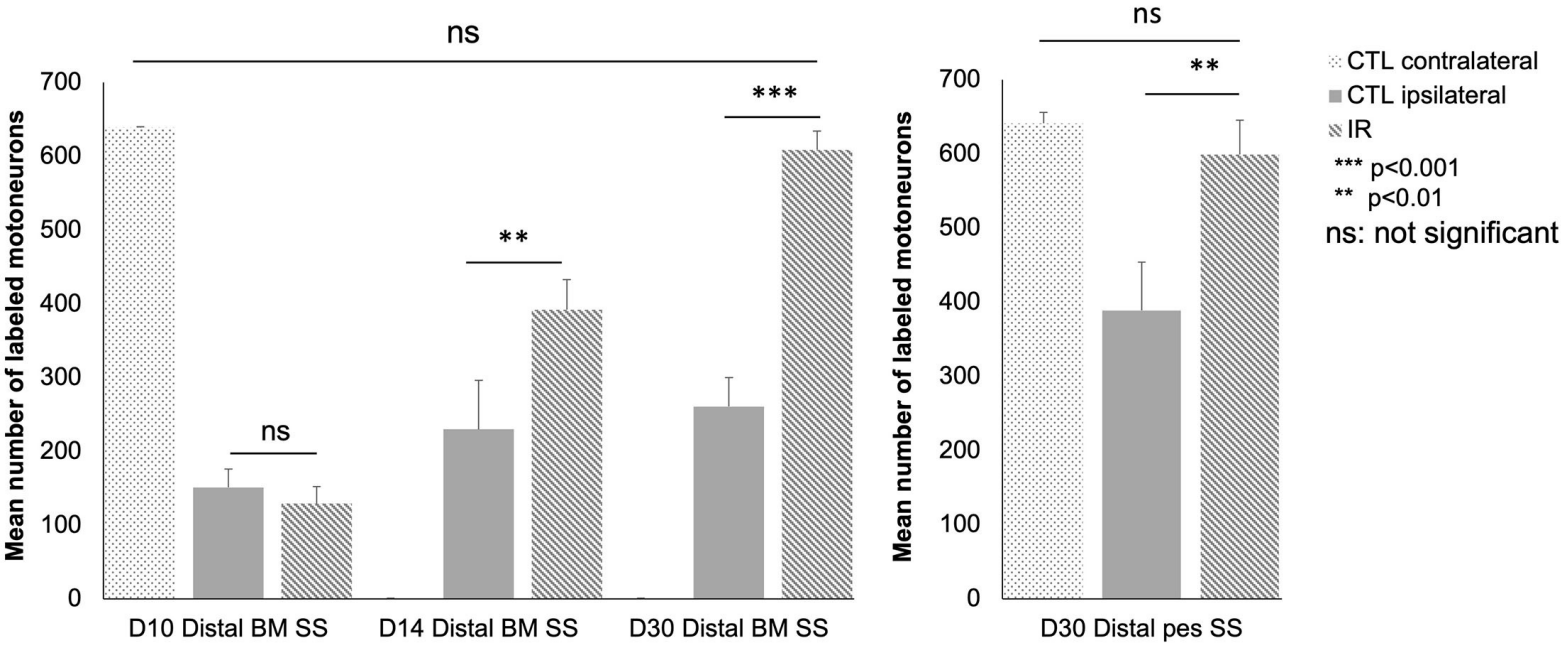
Quantitative analysis of motoneurons in the lateral subnuclei of the facial complex as a function of time after surgery and treatment in the different groups: intact group (*n* = 12), distal BM SS at D10, D14 and D30 control (*n* = 36, 12 animals in each group) and treated animals (*n* = 36, 12 animals in each group), and distal pes SS group at D30 [control (*n* = 6) and treated animals (*n* = 6)]. Note that at D10 only a few motoneurons are labeled. The number of labeled motoneurons increased progressively thereafter: by day 14, the mean number of labeled motoneurons is significantly higher (striped, gray column) in the IR-treated group than in the untreated group (gray column) (*p* < 0.01). The number of labeled motoneurons returned to normal levels on day 30 in the treated mice but not in the untreated mice (*p* < 0.001). Similar results were obtained for the distal pes SS group at D30 between control and treated mice (*p* < 0.001). [*P < 0.05 **P < 0.01 ***P < 0.001 ns *P* > 0.05; *t* test].

We also performed a quantitative analysis (Figure 7). At D10, there was no significant difference between the control and treated mice (distal BM SS: 151.3 ± 25 in control group and 129.3 ± 23 in the IR-treated group; *p* > 0.05).

At D14, a significant difference in the number of retrograde-labeled motoneurons was observed between the control and treated groups (distal BM SS: 230 ± 47.03 in control group and 393.2 ± 40.66 in the IR-treated group; *p* < 0.01).

At D30, the number of retrograde labeled motoneurons was greater in the treated group than in the control group (distal BM SS: 261 ± 38.91 in control and 608.8 ± 25.65 in the IR-treated group; *p* < 0.001). No significant difference was detected between the number of labeled motoneurons on the contralateral side in the control and treated animals (distal BM SS: 620 ± 16.8 in control and 644 ± 20.2 in the IR-treated group; *p* > 0.05). A similar result was obtained for SS of the distal pes at D30 (Distal pes SS: 398.3 ± 56.66 in controls and 588.8 ± 46.32 in IR-treated group). The only difference concerned the number of labeled ipsilateral motoneurons at D30 in the untreated groups (261 ± 38.91 vs. 398 for distal pes SS).

In summary, on D14 and D30, the treated mice had larger numbers of labeled motoneurons than the control mice, for both surgical models. However, the difference in the number of labeled motoneurons between the treated and control mice was much more marked at D30 than at D14.

## Discussion

### Behavioral Effect of IR

In this work, we found a positive effect of PBM in the different models of facial nerve injury. We chose to use a section-suture of the distal buccal, marginal mandibular and proximal buccal branches or of the distal pes, because the branches were superficial (immediately under the skin) and were, therefore, readily accessible for light treatment. This situation contrasts with the proximal facial nerve section model, in which the lesion is created at the exits of the foramen stylomastoid, which are located in a deeper position (presence of subcutaneous fat, blood vessels and muscle tissue), with a possible reduction of total energy of IR or red light into the target areas. In addition, in the proximal facial nerve section model, the functional recovery was not observed before 3 months and very often the recovery was abnormal with synkinesia. Synkinesia were never observed in our peripheral model.

Our results were in accordance with previous studies on the facial nerve: 830 nm IR has been reported to induce functional facial nerve recovery and an increase in the number and density of regenerated axons (18). A significant difference between treated and untreated rats following section-suture of the buccal branch has been demonstrated, with a larger mean nerve fiber area in rats treated by PBM at 830 nm (1).

The effects of PBM therapy have been also widely reported after injury of the sciatic nerve (19, 20). Sciatic functional index (SFI) and range of motion (ROM) analyses have revealed an acceleration of functional nerve recovery following PBM treatment (830 nm) at 8 J/cm^2^ 14 days after surgery (21). PBM (780 nm) has been shown to improve morphometric data for the diameter of nerve fibers, axon diameter, thickness of the myelin sheath and G-ratio 15 days after crushed sciatic nerve injury, but without a return to control levels (22). Following sciatic nerve transection, PBM may preserve the denervated gastrocnemius muscle by maintaining creatine kinase (CK) activity and the number of acetylcholine receptors (AChR) (19, 23). PBM therapy has been shown to increase the expression of neurotrophic factors after sciatic nerve axonotmesis (632, 8 nm). In particular, the levels of nerve growth factor (NFG), brain-derived neurotrophic factor (BDNF), and neurotrophin-3 (NT-3) have been demonstrated to increase 14 days after sciatic nerve crush injury, with peak values obtained at D21 (24). These results are consistent with those of another study (25) showing that NFG and BDNF levels were increased by PBM at 904 nm (by 53% relative to the control for NFG and 40% for BDNF) after alveolar nerve lesion.

However, parameters used such as total energy, power density, type of wavelength were highly controversial in the literature. The method of delivery could also differ depending of the device used to produce PBM laser vs. diode. Therefore, it renders difficult to determine a precise methodology to increase axonal regeneration. Here, we try to precise all the parameters (Table 2) for potential additional therapy in patients suffering from a facial nerve palsy (26).

In addition, in these models, lesion is very often a crush and not a SS. The crush was a very different injury with no Wallerian degeneration and a rapid recovery. For instance, in the facial nerve, a crush of the main branch at the exit from the stylomastoid foramen induced a facial palsy which recovers in about 10 days.

### Molecular Effect of PBM

PBM therapy seems to increase electron availability for the reduction of molecular oxygen, thereby increasing mitochondrial membrane potential (MMP) and the levels of ATP, and cAMP (27) *via* the activation of cytochrome *c* oxidase. This oxidase is a metalloprotein that passes electrons between copper and iron ions. Light stimulates this enzyme, thereby accelerating the production of ATP and physiological free radicals.

Since 2015, several studies have identified water and oxygen as essential targets of light therapy. Water is the most abundant molecule in cells, accounting for at least 70% of the total cell mass. Nanoscopic interfacial water layers (IWL) are attached to surfaces. These layers consist of two to three monolayers of water molecules.

Interaction between the photons provided by light therapy and the IWL has at least two biologically important impacts: changes in IWL density (volume expansion) and decreases in IWL viscosity. The last step in the respiratory chain is mediated by ATPase, acting as a kind of motor, lubricated by the film of water that surrounds it. Light, particularly at near-IR wavelengths (800 to 1,000 nm), decreases the density and viscosity of water, accelerating ATPase turnover and, thus, the production of ATP (28).

### Dynamic Functional Recovery

The recovery at the level of the vibrissae movements was incomplete at day 30 (score 2,5). In contrast, a complete eye closure in treated mice was observed at D20 and a complete disappearance of nasal deviation was found at D16. We hypothesized that the recovery of static (nasal deviation, eye closure) and dynamic (whisker movement) deficits followed different time courses.

Several hypotheses could be proposed to explain the lack of dynamic deficit recovery.

A first factor limiting the restoration of facial function may be the poly-innervated endplates of the muscle after facial nerve section-suture (2, 29). The control of a muscle fiber by at least two asynchronously triggered motor neurons is not physiologically advantageous. One previous study suggested that the proportion of poly-innervated endplates was significantly lower 2 months after facial nerve repair in animals undergoing facial section-suture followed by manual stimulation of the whisker pads on the side ipsilateral to injury (2).

Secondly, a partial central deafferentation of motoneurons may persist. The total number of glutamatergic terminals (VGLUT2+) innervating the facial motoneurons on the injured side after the crushing of a facial nerve at the level of the trunk was significantly smaller than that on the intact contralateral side 2 months after surgery (30). The number of cholinergic terminals (ChAT) on motoneuron cell bodies ipsilateral to injury was also found to be significantly lower, with a positive correlation between the amplitudes of whisking and the smaller number of glutamatergic and cholinergic facial terminals.

Thirdly, the electrophysiological properties of the facial motoneurons may change after injury. Axotomy of the cat facial nerve has been shown to induce a decrease in axonal conduction velocity and a decrease in the duration of after-hyperpolarization following each spike (31). It has been suggested that these changes are due to the modulation of mRNA levels for type I, II and III voltage-sensitive sodium channels in motoneurons following facial nerve transection affecting the genesis of action potentials (32). Levels type I channel mRNA were low and did not begin to increase again until 21 days after surgery. Type III mRNA levels were high between 1 and 3 days after nerve injury, returning to normal on D35. No changes type II sodium channel mRNA levels were observed. These channel changes modify neuronal excitability and the resting of the facial motoneurons after injury.

Other studies (33) have suggested that the fast-twitch EDL (extensor digitorum longus) is selectively impaired after natural reinnervation following sciatic nerve crush injury in adult rats. The myosin heavy chains (MHCs) of the control EDL consisted of about 2% (0–3%) type 1, about 31% (24–36%) type 2A and about 67% (64–72%) type 2B. The normal MHC profile was not restored during EDL muscle recovery after reinnervation, with the persistent loss of a large proportion of the 2B isoform. This pattern persisted for at least 2 months after surgery. Similarly, the myosin heavy chains of lesioned whisker pads in mice may display long-term changes in type 2B isoform levels.

Finally, similar discrepancy between tonic and phasic fibers have been related in others model of sensory deafferentation. For instance, after unilateral labyrinthectomy in rodents, the recovery of the static deficits was complete (ocular nystagmus, head tilt, rolling and circling on the ipsilateral deafferented side) whereas the dynamic deficits never returned to normal (abnormal gain of the vestibulo-ocular reflex at high head velocities) (34). All the data suggested that the tonic sensory or motor pathway may totally recover in contrast to phasic pathways after lesion.

## Conclusion

This study reveals a significant effect of LED-mediated IR exposure on the functional recovery of the facial nerve after section-suture. Additional studies are now required in rodent to analyze the effect on functional recovery of a combination of mechanical stimulation and IR light treatment. The ultimate hope is that PBM could be developed to reduce the number of sequelae (30%) in patients with Bell's palsy through daily manual stimulation combined with PBM. Indeed, sequelae may present even after manual stimulation alone.

## Data Availability Statement

The raw data supporting the conclusions of this article will be made available by the authors, without undue reservation.

## Ethics Statement

The animal study was reviewed and approved by Ethics Committee for Animal Experimentation of the University of Paris in accordance with the requirements of the European Communities Council Directive of September 22, 2010 (2010/63/UE).

## Author Contributions

HE-R, CV, and BL conceived, designed research, and wrote the paper. CV and HE-R performed all the surgery protocols. LB contributed the section about PBM therapy. All authors reviewed the text and approved the final paper.

## Funding

The authors declare that this study received funding from Physioquanta and Qlarité, France. The funder was not involved in the study design, collection, analysis, interpretation of data, the writing of this article or the decision to submit it for publication.

## Conflict of Interest

The authors declare that the research was conducted in the absence of any commercial or financial relationships that could be construed as a potential conflict of interest.

## Publisher's Note

All claims expressed in this article are solely those of the authors and do not necessarily represent those of their affiliated organizations, or those of the publisher, the editors and the reviewers. Any product that may be evaluated in this article, or claim that may be made by its manufacturer, is not guaranteed or endorsed by the publisher.

## Abbreviations

PBM: Photobiomodulation
LED: Light emitted diode
IR: Infrared
CTL: control
J: joules
Distal BM SS: distal section-suture of the buccal and marginal mandibular branches
Distal SS pes: Section-suture of the distal pes
FG: FluoroGold
ATP: Adenosine triphosphate
NO: Nitric oxide
OOM: Orbicularis oculi muscle
PFA: Paraformaldehyde
PBS: phosphate-buffered saline
Gap43: Growth Associated Protein 43
SFI: sciatic functional index
ROM: range of motion
CK: creatine kinase
AChR: acetylcholine receptors
NFG: nerve growth factor
BDNF: brain-derived neurotrophic factor
NT-3: neurotrophin-3
MMP: mitochondrial membrane potential
cAMP: Cyclic adenosine monophosphate
IWL: interfacial water layers
VGLUT2: Vesicular Glutamate Transporter 2
ChAT: Choline acetyltransferase
EDL: extensor digitorum longus
MHCs: myosin heavy chains.
